# Chitosan Nanolayered Cisplatin-Loaded Lipid Nanoparticles for Enhanced Anticancer Efficacy in Cervical Cancer

**DOI:** 10.1186/s11671-016-1698-9

**Published:** 2016-11-25

**Authors:** Jing-yi Wang, Yu Wang, Xia Meng

**Affiliations:** 1 Department of Obstetrics and Gynecology, Second Affiliated Hospital of Chengdu Medical College, No.4, Erhuang Road, Chendong, 610051 Sichuan People’s Republic of China; 2 Department of Health, NYS, Wadsworth, USA

**Keywords:** Cervical cancers, Cisplatin, Antitumor efficacy, Solid lipid nanoparticles, Chitosan

## Abstract

In this study, cisplatin (CDDP)-loaded chitosan-coated solid lipid nanoparticles (SLN) was successfully formulated to treat HeLa cervical carcinoma. The formulation nanoparticles were nanosized and exhibited a controlled release of drug in physiological conditions. The blank nanoparticles exhibited an excellent biocompatibility profile indicating its suitability for cancer targeting. The incorporation of CDDP in SLN remarkably increased the cancer cell death as evident from the MTT assay. Importantly, CDDP-loaded chitosan-coated SLN (CChSLN) significantly (*P* < 0.05) decreased the viability of cancer cells even at low concentration. The higher cytotoxicity potential of CChSLN was attributed to the higher cellular uptake as well as the sustained drug release manner in comparison with CSLN. Consistent with the cytotoxicity assay, CChSLN showed the lowest IC_50_ value of 0.6125 μg/ml while CSLN presented 1.156 μg/ml. CChSLN showed a significantly higher apoptosis in cancer cells compared to that of CSLN and CDDP, which is attributed to the better internalization of nanocarriers and controlled release of anticancer drugs in the intracellular environment. Our findings suggest that this new formulation could be a promising alternative for the treatment of cervical cancers. These findings are encouraging us to continue our research, with a more extended investigation of cellular response in real time and in animal models.

## Background

Human cervical cancer is one of the popular cancers in women in reproductive age across the globe [1]. The cervical cancer is caused by human papillomavirus (HPV). International Agency for Research on Cancer has stated the main factors responsible for the cervical cancers including viral infection, inordinate sexual behavior, depleted immune system, and smoking [2]. Recent advances in cancer diagnosis and use of vaccine have reduced the mortality rate of cervical cancer patients; nevertheless, cancer-related death in China is still high. Chemotherapy, surgery, and radiotherapy are some of the treatment options in cervical cancers; however, none is effective in controlling the cervical cancers [3]. Recently, two prophylactic HPV vaccines (Gardasil and Cervarix) were marketed to tackle HPV-associated cervical cancers; however, it was effective only in adult patients and failed to show any therapeutic effect against present cases. Especially, in the clinical settings, chemotherapeutic drugs play an important role in killing the cervical cancer cells [4, 5].

In this perspective, cis-dichlorodiammineplatinum(II) (cis-[PtCl2(NH3)2], cisplatin (CDDP)) is a potent anticancer agent for the treatment of various solid cancers including cervical cancers [6]. CDDP kills the cancer cells by crosslinking the DNA which in turn results in the cellular apoptosis as the repair become unsuccessful. The potent anticancer effect of CDDP is hindered by its severe adverse effects such as nephrological and neurological toxicities [7]. CDDP is normally associated with nephrotoxicity that results in acute and chronic morbidity, while neurotoxicity is cumulative-dose dependent. More than everything, one of the biggest worries is the immediate inactivation of CDDP in the systemic circulation that will reduce its therapeutic potency and results in unwanted side effects [8, 9]. Therefore, efforts have to be made to protect the pharmacological action of CDDP in systemic circulation and prolong its systemic circulation. Furthermore, drug has to be released in a sustained or controlled manner that will improve its anticancer effect and reduce its associated side effects.

The applications of principles of nanotechnology in cancer treatment are expected to solve all the existing problems. The drug loaded in a nanocarrier could effectively protect the exposure of chemotherapeutic drug to the extracellular environment [10]. Moreover, physiological conditions of the diseased cancerous tissue offer many benefits to the delivery system. The physiological changes in the tumor tissue could be effectively used to passively target the drug in the tumor. The poor lymphatic drainage and increased vascular permeability in cancer cells allows the drug-loaded nanoparticle to accumulate passively in the tumor via enhanced permeation and retention (EPR) effect. Additionally, nanoparticle will offer multiple advantages such as increased stability, high loading capacity, sustained release of drugs, and minimize the drug-related side effects [11, 12].

**Fig. 1:**
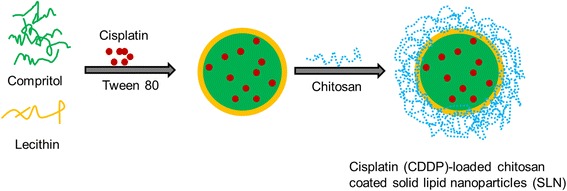
Graphical presentation of the preparation of cisplatin-loaded chitosan-coated solid lipid nanoparticles

In this regard, solid lipid nanoparticles (SLN) are reported to be potential drug carriers. SLN provides some of the unique benefits such as small particle size, high surface area, spherical shape, and in vivo stability that have generated huge enthusiasm in pharmaceutical and biomedical enterprises [13–15]. Especially, SLN possess the benefits of normal nanocarriers including polymeric and lipid nanoparticles, simultaneously minimize its drawbacks. The SLN carrier better protect the encapsulated drugs in the biological circumstances and offer better physiochemical features. However, one of the main drawbacks of SLN is its inability to control the drug release in the systemic conditions [16, 17]. Therefore, effective strategies have to be made to prevent the drug release and enhance the physicochemical properties of SLN. Surface modification or assembly of biological polymer coating could be employed to develop sustained release characteristics SLN. Chitosan, non-toxic, biodegradable, and biocompatible polysaccharide could be an ideal choice to coat the SLN surface. The chitosan-coated SLN is expected to act as a promising vehicle for the delivery of CDDP in cervical cancers [18]. In the present study, a biocompatible chitosan was employed for surface modification which will act like a stealth layer (Fig. 1).

In this work, we primarily aimed to prepare chitosan-coated SLN to effectively deliver CDDP in cervical cancers. Towards this aim, drug-loaded nanoparticle was characterized in terms of particle size, zeta potential, morphology, and release characteristics. The anticancer effect of free CDDP and CDDP-loaded SLN was investigated in HeLa cervical cancer cells.

## Methods

### Materials

Compritol 888 ATO and monooleate of sorbitan ethoxylated (Super refined Polysorbate 80TM; Tween 80TM) were kindly provided by Gattefossé (Saint Priest, France). Cisplatin and chitosan was purchased from Sigma-Aldrich, China. All other chemicals were of analytical grade and used as such.

### Preparation of Cisplatin-Loaded Chitosan-Coated Solid Lipid Nanoparticles

The SLN was formulated by hot homogenization followed by emulsification-ultrasound method. Briefly, 100 mg of Compritol, 10 mg of lecithin, and 10 mg of CDDP were melted above 60 °C and constitutes the oil phase. The aqueous phase consists of Tween 80 (2%), and the temperature was maintained similar to that of oil phase. While maintaining the same temperature, surfactant-containing phase was added slowly to the oil phase under constant agitation. The mixture was immediately homogenized using Ultra Turrax T-25 homogenizer for 5 min. The homogenized solution was immediately subjected to high intensity probe sonication (Vibracell VCX130; Sonics, USA) for 5 min. The emulsion was immediately cooled to 4 °C to allow the SLN formation. For chitosan coating, 0.1% chitosan solution was prepared in 0.1% acetic acid. The SLN and chitosan solution was mixed and stirred for 2 h at 100 rpm. The chitosan-coated SLN was separated and lyophilized (if necessary) and stored.

### Dynamic Light Scattering Analysis

The size distribution was analyzed by dynamic light scattering technique using BI-200SM, Brookhaven Instruments Corp., Holtsville, NY, USA, equipped with a 35-mM HeNe laser beam at a wavelength of 637 nm. The zeta potential of nanoparticle was analyzed using laser Doppler velocimeter (Zetasizer Nano ZS90, Malvern, UK).

### Drug Encapsulation Efficiency and Drug Loading

The CDDP-entrapped nanocarriers were centrifuged at high speed (15,000 rpm), and the supernatant was collected to evaluate the amount of unloaded drugs. HPLC method was used to quantify the drug loading and entrapment efficiency. HPLC (Agilent LC 1100, Santa Clara, CA, USA) was used. The column consists of Inertsils ODS-3 column (150 4.6 μm, pore size 5 μm, GL Science Inc., Tokyo, Japan) was used.

### In Vitro Drug Release

Dialysis method was used to perform the release study. Briefly, 1 ml of drug-loaded nanoparticles was packed in a dialysis membrane (Spectra/Por 6, MWCO 3000, Spectrum Laboratories, Inc., TX, USA) and both the ends were sealed. The dialysis membrane was sealed and kept in 20 ml of release buffer and in turn placed in a shaker bath at 100 rpm. At specific time point, 1 ml of release buffer was taken out and replenished with same amount of new medium. The concentration of drug in the release medium was quantified by HPLC method. The mobile phase used was methanol/water/acetonitrile (40:30:30 *v*/*v*/*v*) at 1 ml/min.

### Cytotoxicity Assay

DMEM medium was used to grow HeLa cell which is supplemented with 10% of FBS and 1% of antibiotic mixture in an incubator at 37 °C. The cytotoxicity assay was performed by MTT protocol. Briefly, 15,000 cells/well was seeded at each well of 96-well plate and let it aside for 24 h. The old media was removed and wells containing adherent cells were washed with PBS. The cells were incubated with SLNs (0.01–100 μg/mL) for 24 h. Also, free CDDP and CDDP-loaded formulations were exposed to cancer cells and incubated for 24 h. Following 24-h incubation, the old media containing free SLN and drug-loaded formulations were carefully aspirated and the cells were washed twice with PBS. This process was carried out in order to minimize the chance of carrier or drug that is not internalized by the cells and avoid any interference of the materials on the final absorbance. The cells were then added with 10 μl of MTT (5 mg/ml) and incubated for 4 h. Then MTT solution was removed or aspirated carefully, and cells were added with 150 μl of DMSO to solubilize the formazan crystals. The well plates were then kept aside for 15 min, and absorbance was studied at 570 nm using a POLAR star microplate reader (Omega, BMG LabTech). The IC_50_ value was calculated from using GraphPad prism software.

### Apoptosis Assay

The apoptosis assay was performed by Annexin V/PI staining. In brief, cells (2 × 10^5^) were seeded in 12-well plate and left aside for 24 h. The cells were treated with respective formulations and left untouched for 24 h. The cells were washed, isolated, and treated with Annexin V and PI dye for 15 min in dark conditions. The cells were then resuspended in PBS and studied using flow cytometer (BD Biosciences, USA).

### Statistical Analysis

Analyses for the cytotoxicity studies were performed using one-way ANOVA followed by Tukey’s test. *P* value <0.05 was considered statistically significant for all analyses.

## Results and Discussion

### Physicochemical Characterization of CChSLN

**Fig. 2:**
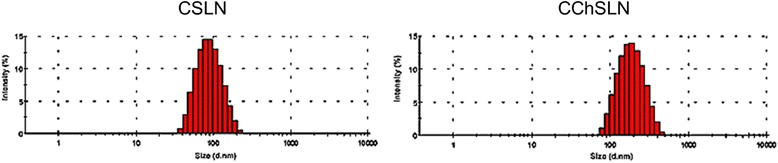
Particle size distribution of CSLN and CChSLN. The particle size of nanoparticles was measured by dynamic light scattering (DLS) technique

SLN was prepared by hot homogenization followed by emulsification-ultrasound method. Among all lipids, we have selected Compritol owing to its high loading capacity and high bioavailability of encapsulated drugs. CDDP-loaded SLN (CSLN) was prepared with an average size of ~110 nm with an excellent dispersity index. The final particle size after chitosan coating becomes ~190 nm (Fig. 2). The particle size increased due to the assembly of solid chitosan on the outer surface of SLN. Furthermore, the CSLN which we prepared possessed a negative ZP (approximately −26 mV) charge. The functional groups present on the outer surface of NP led to the negative charge. The surface charge of CSLN reversed to +22 mV after chitosan coating indicating the presence of solid complimentary material on the nanocarrier surface. It has been known that charge present on the nanoparticle surface will decide its fate in vivo. The positively charged nanoparticles will be beneficial as the positively charged particle will interact favorably with the cells containing negative charge [19]. It has been reported that the small size of particle will be beneficial for the enhanced internalization of drugs via endocytosis. It has been reported that SLN of particle size between 80 and 300 nm could be internalized by various endocytosis pathways in cancer cells. Moreover, no significant change in particle size of CChSLN was observed when stored over the period of 5 weeks (data not shown). The results clearly reveal the excellent stability of SLN during cell culture investigations. Furthermore, it would be nice if the size of SLNs prepared is small as nanosized particles are more suitable for biomedical applications.

### In Vitro Drug Release Kinetics

**Fig. 3:**
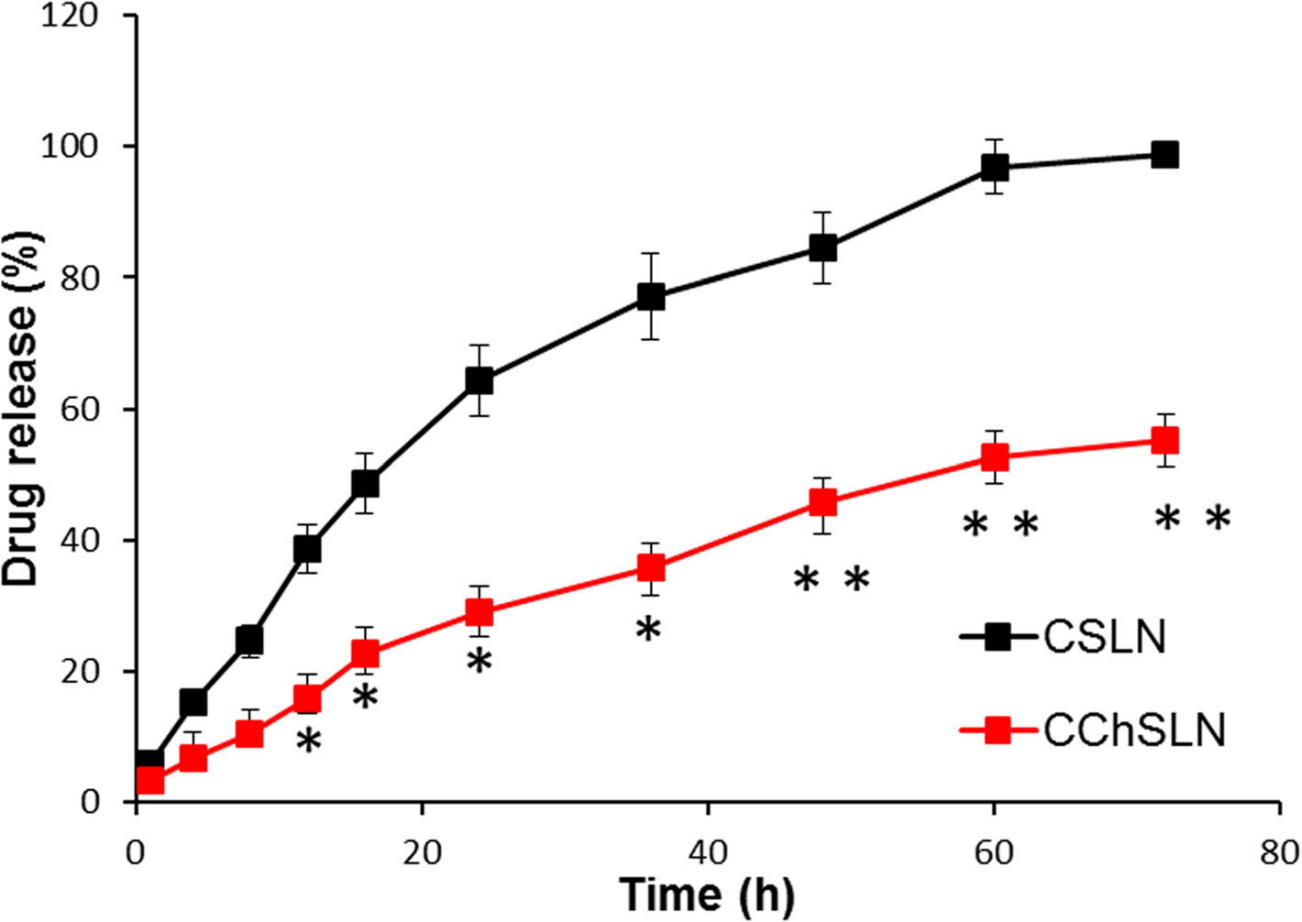
In vitro drug release profile of CSLN and CChSLN. The release study was performed in phosphate-buffered saline (PBS) at 37 °C for 72 h

The release of drug from CSLN and CChSLN was investigated. The phosphate-buffered saline (pH 7.4) was used to simulate physiological conditions to carry out release study (Fig. 3). As expected, release profile of CSLN and CChSLN differed from each other. For example, a typical biphasic release trend was observed in CSLN where in approximately ~25% of CDDP was released within first 10 h of release study while remaining ~75% of drug released in 72 h. In contrast, a well-controlled release of CDDP was observed in CChSLN throughout the release study. Approximately, ~50% of drug was released by the end of the release study. A significant decrease in the release of drug was observed after chitosan coating indicating the importance of presence of stealth layer or protective layer. The decrease in the release rate was attributed to the increase in the path length of drug from the core of the nanoparticle to the outer release media. It has been reported that the solid status of the SLN at body temperature and strong hydrophobic interaction of drug with lipid nanoparticles is the main reason for the controlled release of drug.

### Cellular Uptake Analysis

**Fig. 4:**
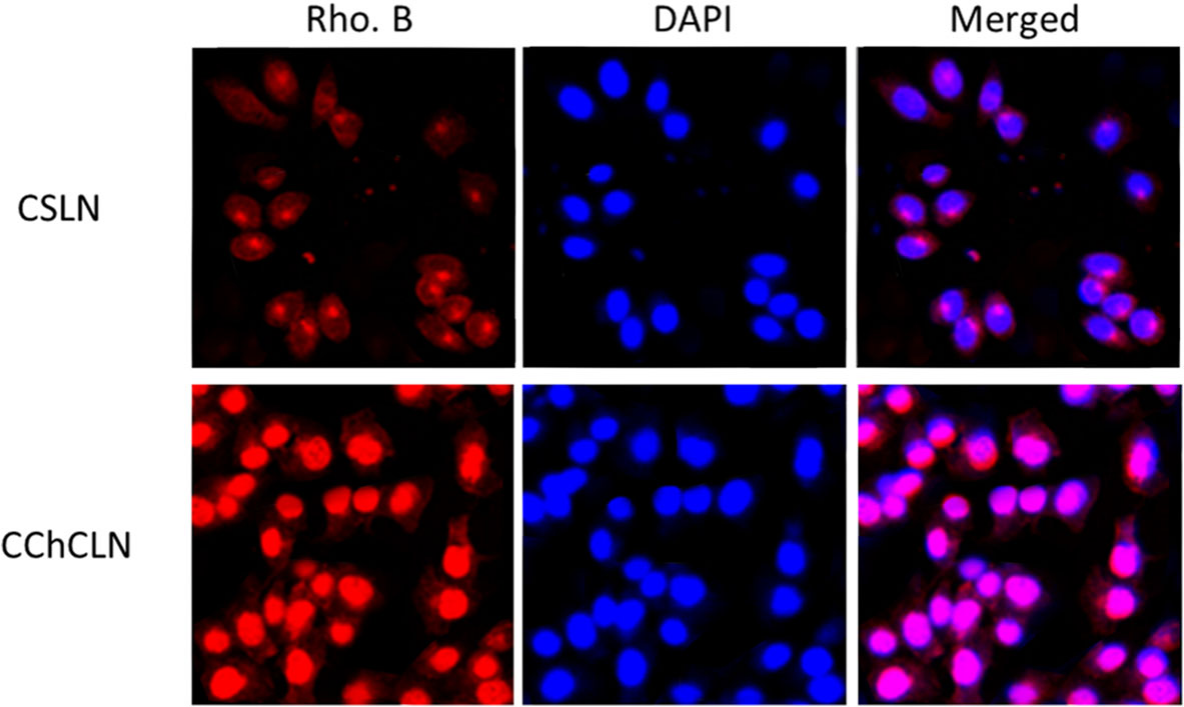
In vitro cellular uptake of CSLN and CChSLN in HeLa cancer cells. Rhodamine B was used as a fluorescent probe

The cellular uptake of CSLN and CChSLN was performed by confocal laser scanning microscopy (CLSM) (Fig. 4). As seen, CChSLN showed a remarkably higher uptake in cancer cells compared to that of CLSN. The marked cellular uptake of CChSLN in cancer cell was attributed to the cationic surface charge of nanoparticles that was internalized on the negatively surface charged cellular membrane.

### Biocompatibility of Blank Nanoparticles

**Fig. 5:**
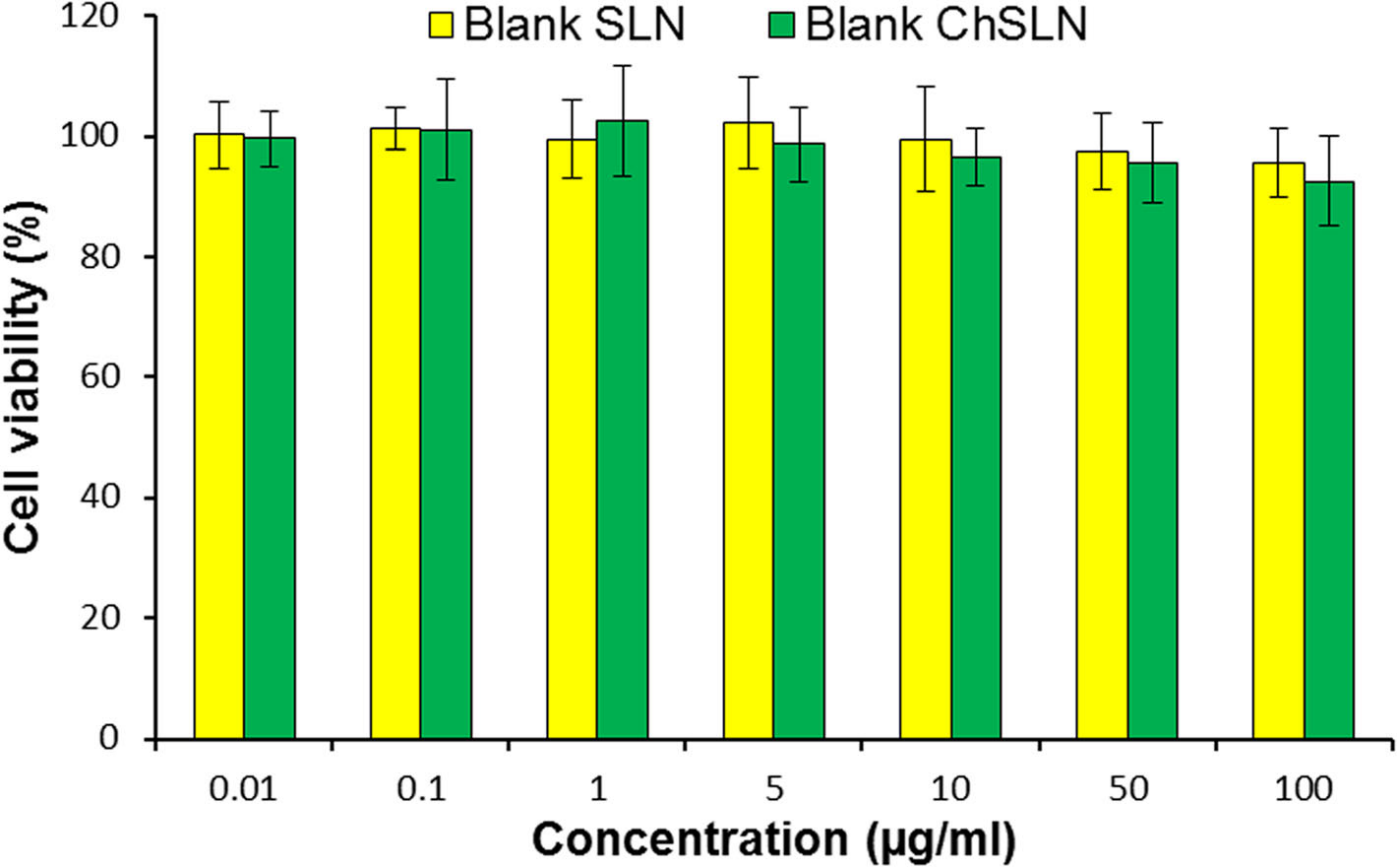
Biocompatibility analysis of blank SLN and chitosan-coated SLN in HeLa cervical cancer cells

The biocompatibility of SLN is one of the important criteria for the successful application of nanocarrier in cancer targeting. As shown, both the blank nanoparticles did not affect the growth of cancer cells (Fig. 5). Especially, cell viability remained more than >95% even when treated highest concentration of blank SLN while the cell viability remained more than >90% in case of chitosan-coated SLN. A slight decrease in the cell viability of chitosan-coated formulation was due to the positive charge of chitosan polymer which may kill the cancer cells sometimes. Overall, high cell viability warrants the safety profile and biocompatibility of SLN formulations.

### Cytotoxicity Assay of Free CDDP and CChSLN

**Fig. 6:**
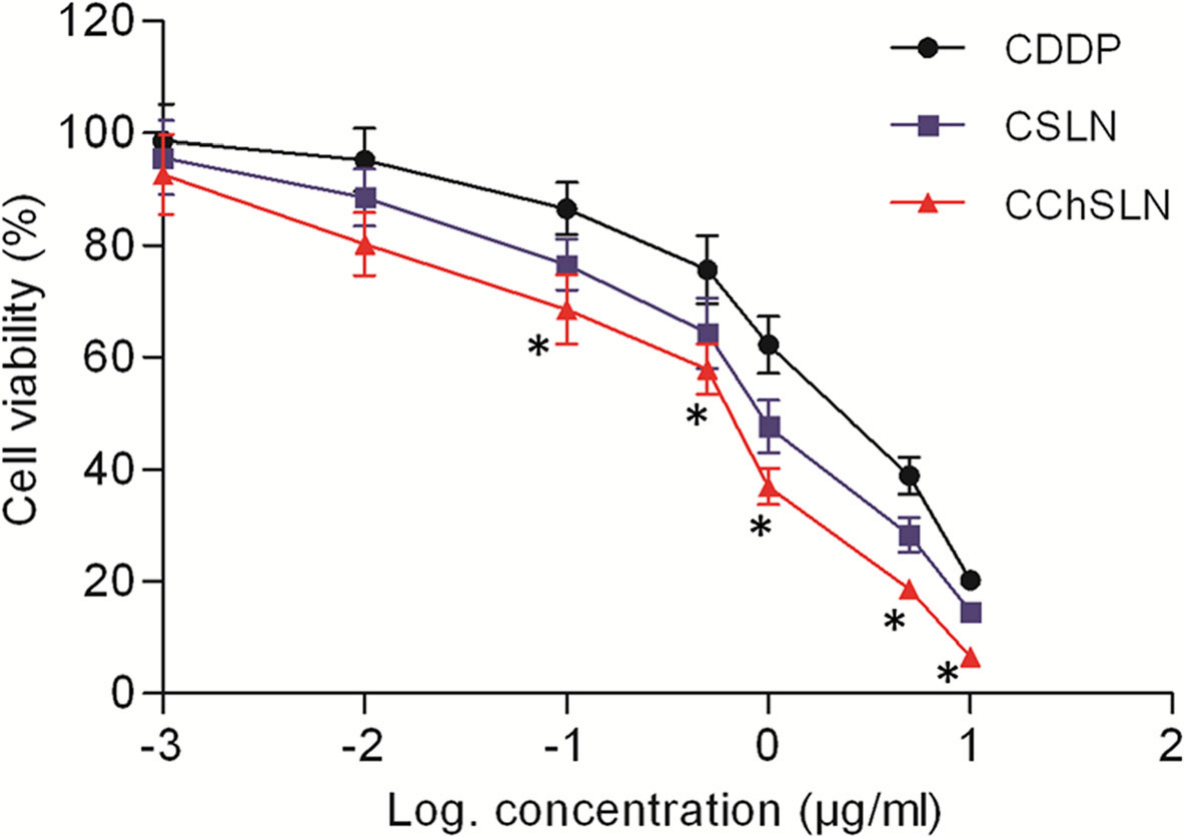
Cytotoxicity potential of CDDP, CSLN, and CChSLN in HeLa cervical cancer cells. The cytotoxicity of formulations was analyzed by MTT assay

For the successful treatment of cervical cancers, it is utmost important to improve the chemotherapeutic efficacy of CDDP while reducing its side effects on the normal cells. Towards this goal, we have applied the nanotechnology principles to improve the biological and pharmacological effects of CDDP at low concentrations (Fig. 6). The cytotoxicity of CDDP, CSLN, and CChSLN was investigated in HeLa cervical cancer cells using MTT method. The results indicate that the viability of cancer cells were typically dependent on the concentration of drug or drug-loaded formulations. The free CDDP although reduced the cell viability of cancer cells, it was not very effective. As expected, incorporation of CDDP in SLN remarkably increased the cancer cell death as evident from the MTT assay. Importantly, CChSLN significantly decreased the viability of cancer cells even at low concentration. The higher cytotoxicity potential of CChSLN was due to the better internalization in the cancer cells and controlled release of encapsulated component in comparison with CSLN. The positively charged CChSLN is expected to internalize in higher rate and increase the concentration gradient of CDDP in the cancer cells. In addition, nanoparticles greatly help minimize the multidrug resistance which is responsible for the elimination of many anticancer drugs by a unique p-glycoprotein.

**Fig. 7:**
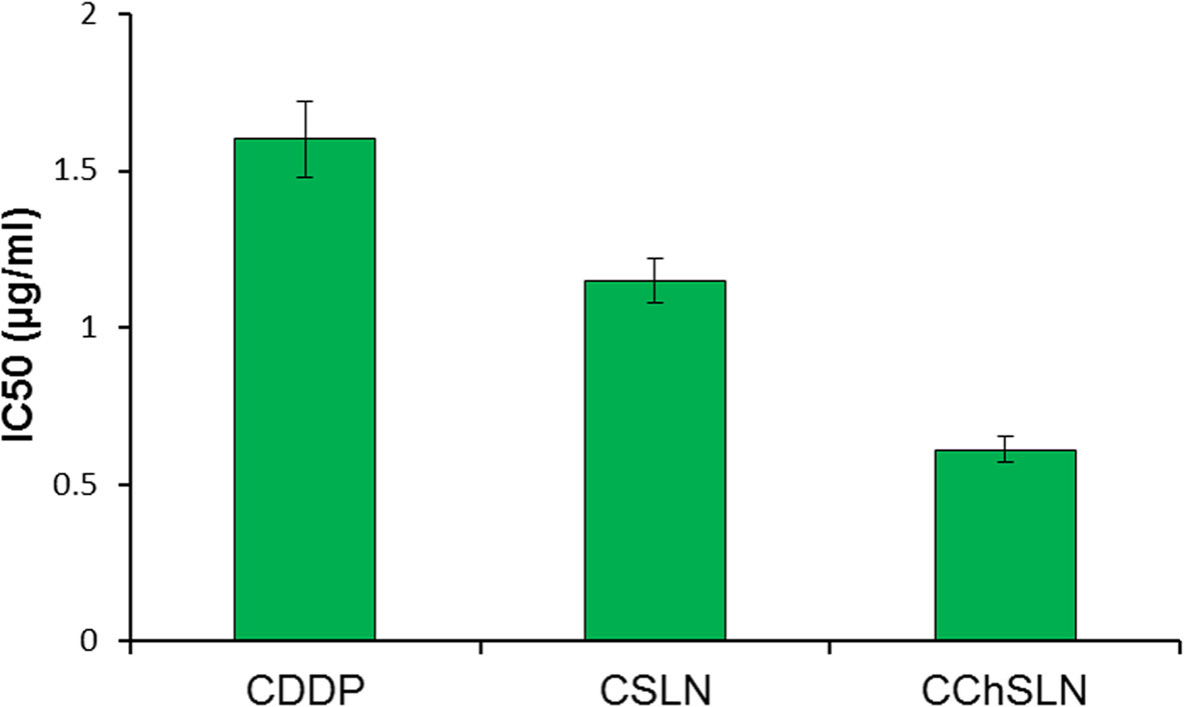
IC_50_ value of CDDP, CSLN, and CChSLN in HeLa cervical cancer cells

The antitumor effect of different formulations was further quantified by IC_50_ value, which is defined as the amount of drug required to kill 50% of cells in the designated time interval (Fig. 7). Consistent with the cytotoxicity assay, CChSLN showed the lowest IC_50_ value of 0.6125 μg/ml while CSLN presented 1.156 μg/ml. The CDDP showed an IC_50_ value of 1.602 μg/ml which is highest among all the formulations tested. The data clearly show the advantage of the nanoparticle formulation vs. free CDDP.

### Microscopic Analysis

**Fig. 8:**
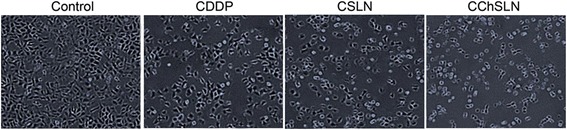
Morphological analysis of cancer cells after treatment with CDDP, CSLN, and CChSLN

The cancer cells were treated with individual items and incubated for 24 h. The morphology of cancer cells was consistent with the cytotoxicity data. As shown, CChSLN-treated cells were fewer and round morphology indicating the higher cytotoxicity of the formulations (Fig. 8). The untreated cells were normal and spread over the six-well plate whereas formulation treated group clearly showed the shrinkage and apoptosis of cancer cells. Results clearly reveal that chitosan-coated NP effectively kills the cancer cells and could be beneficial for the cancer treatment.

### Apoptosis Analysis

**Fig. 9:**
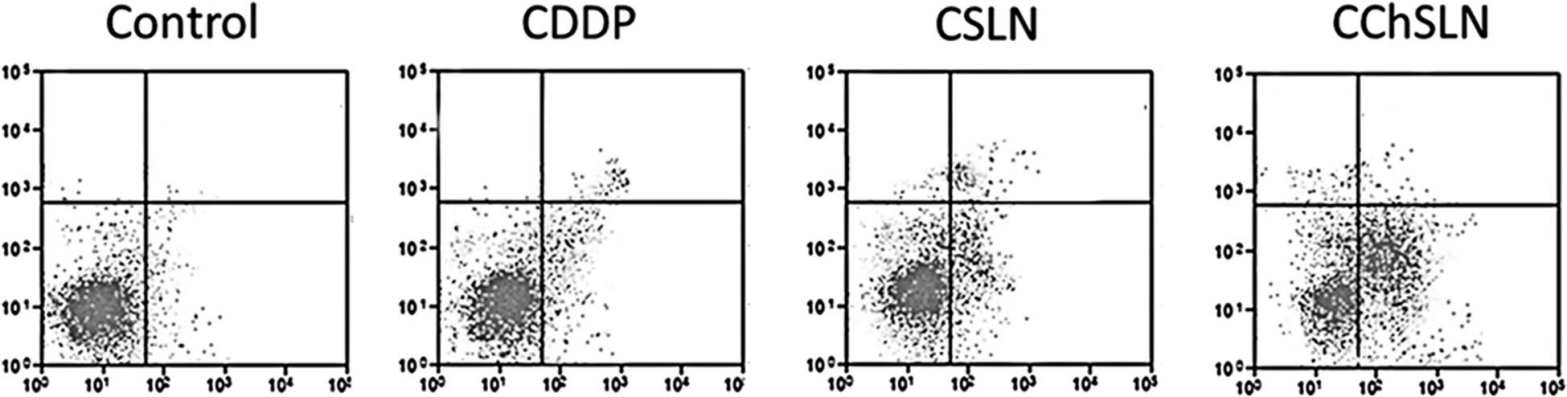
The apoptosis analysis of formulations in cancer cells. The apoptosis assay was carried out by Annexin V/PI staining and determined by flow cytometer

The apoptosis analysis was carried out by Annexin V/PI staining assay. As seen, free CDDP slightly induced an apoptosis of cervical cancer cells while CSLN exhibited a relatively higher cellular apoptosis in this cancer cells (Fig. 9). As expected, CChSLN showed a significantly higher apoptosis in cancer cells which is attributed to the better internalization of nanocarriers and controlled release of anticancer drugs in the intracellular environment. The CChSLN showed nearly ~40% of cell apoptosis compared to that of ~28 and 12% for CSLN and CDDP, respectively.

## Conclusions

In conclusion, CDDP-loaded chitosan-coated SLN was successfully formulated to treat HeLa cervical carcinoma. The formulation nanoparticles were nanosized and exhibited a controlled diffusion of drug in neutral conditions. The blank nanoparticles exhibited an excellent biocompatibility profile indicating its suitability for cancer targeting. The incorporation of CDDP in SLN remarkably increased the cancer cell death as evident from the MTT assay. Importantly, CChSLN significantly decreased the viability of cancer cells even at low concentration. The higher cytotoxicity potential of CChSLN might be due to that enhanced cellular internalization and controlled release of encapsulated component in comparison with CSLN. Consistent with the cytotoxicity assay, CChSLN showed the lowest IC_50_ value of 0.6125 μg/ml while CSLN presented 1.156 μg/ml. CChSLN showed a significantly higher apoptosis in cancer cells compared to that of CSLN and CDDP, which is attributed to the better internalization of nanocarriers and controlled release of anticancer drugs in the intracellular environment. Our results clearly reveal that these unique formulations could be a better choice for cervical cancer treatment. The investigative findings encourage us to carry out in-depth biological and in vivo analysis of the present system.
